# Analysis of channel uncertainty in ARQ relay networks

**DOI:** 10.1371/journal.pone.0190622

**Published:** 2018-01-29

**Authors:** Hina Ajmal, Aimal Khan, Saad Rehman, Farhan Hussain, Mohammad Alam, Rupert Young

**Affiliations:** 1 National University of Sciences and Technology (NUST), Islamabad, Pakistan; 2 Texas A & M University, Kingsville, Texas, United States of America; 3 University of Sussex Sussex House, Falmer Brighton, BN1 9RH, United Kingdom; Beijing University of Posts and Telecommunications, CHINA

## Abstract

Several power allocation algorithms for cooperative relay networks are presented in the literature. These contributions assume perfect channel knowledge and capacity achieving codes. However in practice, obtaining the channel state information at a relay or at the destination is an estimation problem and can generally not be error free. The investigation of the power allocation mechanism in a wireless network due to channel imperfections is important because it can severely degrade its performance regarding throughput and bit error rate. In this paper, the impact of imperfect channel state information on the power allocation of an adaptive relay network is investigated. Moreover, a framework including Automatic Repeat reQuest (ARQ) mechanism is provided to make the power allocation mechanism robust against these channel imperfections. For this framework, the end-to-end SNR is calculated considering imperfect channel knowledge using ARQ analytically. The goal is to emphasize the impact of imperfect channel knowledge on the power allocation mechanism. In this paper, the simulation results illustrate the impact of channel uncertainties on the average outage probability, throughput, and consumed sum power for different qualities of channel estimation. It is shown that the presented framework with ARQ is extremely robust against the channel imperfections.

## 1 Introduction

Cooperative relay networks have recently attracted a considerable amount of research attention over the last decade. These networks have the ability to mitigate the effects of fading channels and provide efficient communication between nodes with poor link quality. In relay networks different forwarding techniques at the relay such as amplify-and-forward (AF), decode-and-forward (DF) [1], [2], and decode-and-forward with soft information relaying [3] have been investigated. AF suffers from the problem of noise amplification, on the other hand DF is deteriorated by error propagation. In order to mitigate these problems the adaptive relaying idea was presented in [4]. In that work, the relay switched from AF to DF and from DF to AF, depending on the decoding status at the relay. This relaying method takes the advantages of both AF and DF and minimizes their disadvantages. This technique was named as adaptive Amplify-or-Decode and Forward. The signal transmission takes place in two phases. In the first phase the source transmits to the relays as well as to the destination. In the second phase all the relays forward irrespective of the decoding status at the destination.

It is obvious that a relay should cooperate only if the destination needs its cooperation, otherwise resources will be used in vain. In [1], a protocol named incremental relaying is introduced in which a relay cooperates if the destination requests its cooperation, the concept of incremental relaying can be viewed as an extension of Automatic Repeat reQuest (ARQ) to the relay context.

Furthermore, due to the limited transmission power of the source and relays, power efficiency is a critical design consideration for wireless relay networks. Several power allocation techniques regarding relay networks are introduced in the literature. Relay forwarding strategies for parallel relay channels in the wideband regime were proposed in [5], the sum power was minimized to achieve a specific rate for amplify-and-forward and decode-and-forward separately. In [6], distributed power allocation strategies with limited channel state information only for decode-and-forward relay network are investigated. In [7], the authors allocate the power by minimizing the outage probability of the system under sum power constraint, considering again only the relays with successful decoding.

Power allocation with adaptive relay network has been introduced in [8]. This reference allocates the minimum power to the nodes such that successful decoding is ensured at the destination.

All of these contributions consider perfect channel knowledge with the assumption of an ideal coding scheme. An ideal code is a code with infinite code length which guarantees successful decoding at the receiver if the respective link capacity is greater than or equal to the effective code rate.

It should be emphasized that the power allocation schemes provided in the above cited contributions are based on the assumption that the channel state information are perfect. Thus, these provide optimal performance only with perfect channel information. However, obtaining the channel state information at a relay or at the destination is an estimation problem and can generally not be error free. Therefore, these estimation errors can deteriorate the performance of a wireless network and can have adverse consequences. These include degraded channel capacity, reduced throughput, and high bit error rate. For example, imperfect channel knowledge and its impact on the capacity of a general wireless single link channel is discussed in [9, 10]. From these contributions, it is clear that channel estimation errors can degrade the channel capacity and the performance of a wireless system significantly. The effect of channel estimation errors on the bit error rate (BER) of an AF relay network is presented in [11]. It shows that even small channel estimation errors can cause up to 2 dB of BER degradation. Therefore, it is worthwhile to investigate and analyze the impact of imperfect channel state information (iCSI) on the power allocation in an adaptive Amplify-or-Decode and Forward relay network. Moreover, it is also important to devise a power allocation framework which takes into account the channel estimation imperfections and are robust to these imperfections.

Imperfect channel knowledge in the context of cooperative relay network has been analyzed in detail for different scenarios and cases, e.g. the analysis presented in [12–16]. However, these papers have their limitations: [12] and [13] investigates a simple AF relay networks for error rate performance and relay selection respectively in the presence of imperfect channel knowledge. These papers have not considered an adaptive relay network or power allocation for the relay nodes. Moreover, a cognitive cooperative sensor network with AF relays are examined for the outage analysis in [16]. This paper considers power allocation but without adaptive relays. Furthermore, the work in [14] discusses a relay network with adaptive rate and imperfect channel state information (ICSI). The main focus of this mentioned work is to select optimal relays in the presence of ICSI and adaptive modulation. Again, it does not consider power allocation with adaptive relays. Finally, all these contributions do not provide a framework to make the power allocation robust to channel imperfections. Thus, we need to address these limitations. Our contribution in this manuscript is that we have investigated the ICSI in adaptive relay network with power optimization procedure presented in [8]. Moreover, a framework is presented based on ARQ to make the power allocation schemes robust to these imperfections.

The rest of the paper is organized as follows. Sec. 2 presents the system model under consideration. In Sec. 3, the concept of ideal coding and different relaying protocols are discussed. The summary of a power allocation algorithm assuming ideal coding with perfect channel knowledge is discussed in Sec. 5. Sec. 7 describes the end-to-end performance of an ARQ adaptive relay network with channel estimation errors.

Sec. 6 introduces different ARQ strategies with imperfect channel knowledge utilizing the power allocation algorithm discussed in Sec. 5. Finally, Sec. 8 discusses the simulation results, while, Sec. 9 concludes the paper.

## 2 System model

**Fig 1:**
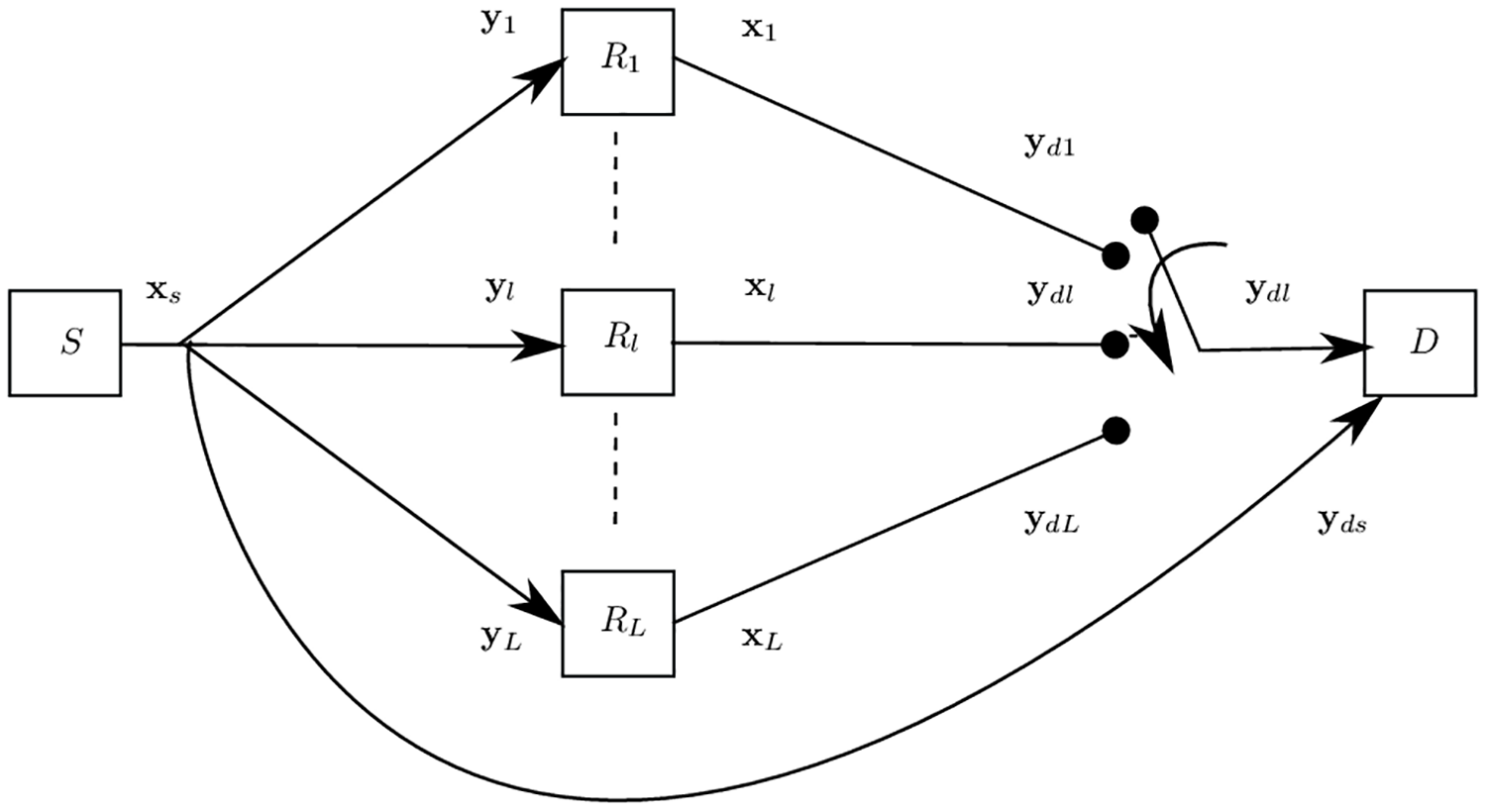
System model showing one source *S*, multiple relays *R*_*l*_ and one destination *D* with respective channels and signals.

The system model in Fig 1 is restricted to a dual-hop parallel relay network, where, *L* relays are placed between source *S* and destination *D*. The relay set is denoted by R. The basic Parallel Relay Network was introduced for the first time by Gallager in [17] and has been studied in [5, 6, 18, 19]. In this sort of network, relays benefit from the transmissions of a source, but not from other relays transmissions.

The source encodes its information bits using arbitrary encoding and modulation. The channel from the source to the *l*th relay $R_{l}\in R$ is parameterized by $h_{ls}^{\prime }$, *a*_*ls*_, and $\sigma_{n}^{2}$, where 1 ≤ *l* ≤ *L*. Here, $h_{ls}^{\prime }$ is assumed to be an i.i.d. Rayleigh distributed block-fading coefficient with $E\{{\left(h_{ls}^{\prime }\right)}^{2}\}=1.$ Moreover, $a_{ls}=\sqrt[{}]{{\left(d_{ls}\right)}^{-a}}$ represents the path loss factor for source-relay distance *d*_*ls*_ with path loss exponent *a*. Each element of the complex noise vector **n**_*ls*_ is from an AWGN process with mean zero and variance $\sigma_{n}^{2}$. The received baseband signal at the *l*th relay from the source is given by

$$\begin{array}{c} y_{l}=h_{ls}\cdot \sqrt[{}]{\alpha_{s}}\cdot x_{s}+n_{ls}, \end{array}$$ (1)

with $h_{ls}=h_{ls}^{\prime }\cdot a_{ls}$ holds and *α*_*s*_ is the power allocated to the source. At the destination the signal received from the source through the direct path is given by

$$\begin{array}{c} y_{ds}=h_{ds}\cdot \sqrt[{}]{\alpha_{s}}\cdot x_{s}+n_{ds}, \end{array}$$ (2)

with $h_{ds}=h_{ds}^{\prime }\cdot \sqrt[{}]{{\left(d_{ds}\right)}^{-2}}$. Furthermore, $h_{ds}^{\prime }$ and **n**_*ds*_ have the same statistics as $h_{ls}^{\prime }$ and **n**_*ls*_ respectively, and *d*_*ds*_ represents the distance between the source and the destination.

The *l*th relay processes the received signal **y**_*l*_ and transmits the signal **x**_*l*_. It is assumed that all the nodes transmit in an orthogonal time slots. At the destination, the equivalent received baseband signal from the *l*th relay can be written as

$$\begin{array}{c} y_{dl}=h_{dl}\cdot x_{l}+n_{dl}, \end{array}$$ (3)

having identical AWGN and fading channel statistics as in (2) but the path loss depends on the distance from the *l*th relay to the destination. The destination performs maximum ratio combining of all the received signals from the source and the relays to decode the signal transmitted from the source.

## 3 Error control coding

**Fig 2:**
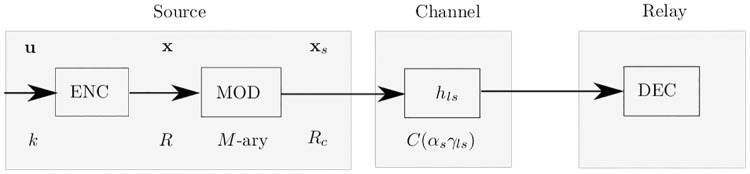
Encoding, modulation, and decoding.

In this section, ideal coding scheme as error control coding scheme is discuessed. A source-relay link highlighting encoding, modulation, and decoding is shown in Fig 2. The source encodes *k* bits long information sequence **u** to a coded sequence **x** of length *n* with code rate *R*_*c*_. Afterwards, sequence **x**_*s*_ is generated by applying arbitrary *M*-ary modulation with modulation size *m* = log_2_(*M*) and transmit power *α*_*s*_. The effective rate after modulation is denoted by *R* = *m* ⋅ *R*_*c*_. The modulated sequence is transmitted over the channel with capacity *C*(*α*_*s*_ ⋅ *γ*_*ls*_), where, *α*_*s*_ ⋅ *γ*_*ls*_ is the SNR of the link from the source to the *l*th relay. At the relay, the respective decoding is performed.

A coding scheme of rate *R* is called an ideal coding scheme for a communication channel of capacity *C*(*α*_*s*_ *γ*_*ls*_) if it minimizes the bit error probability [20]. According to Shannon’s channel coding theorem, completely error free communication is only possible if the capacity *C*(*α*_*s*_ ⋅ *γ*_*ls*_) of a channel is larger than or equal to the effective code rate *R* and *n* approaches ∞.

Therefore, ideal codes deliver completely error free communication, when the condition *R* ≤ *C*(*α*_*s*_ ⋅ *γ*_*ls*_) is satisfied. These codes are parameterized by two variables, 1) Effective code rate *R*, 2) *M*-ary channel capacity *C*(*α*_*s*_ *γ*_*ls*_).

The decoding status for these codes at the relay can be represented as

$$\begin{array}{c} \text{Decoding}\;\text{Status}=\{\begin{array}{cc} \text{Successful}\;\text{Decoding} & \text{for}\;C(\alpha_{s}\cdot \gamma_{ls})\geq R\\ \text{Decoding}\;\text{failure} & \text{for}\;C(\alpha_{s}\cdot \gamma_{ls})<R \end{array}\;. \end{array}$$ (4)

for *R* = *C*(*γ*_*th*_), the above conditions can also be expressed as

$$\begin{array}{c} \text{Decoding}\;\text{Status}=\{\begin{array}{cc} \text{Successful}\;\text{Decoding} & \text{for}\;\alpha_{s}\cdot \gamma_{ls}\geq \gamma_{th}\\ \text{Decoding}\;\text{failure} & \text{for}\;\alpha_{s}\cdot \gamma_{ls}<\gamma_{th} \end{array}\; \end{array}$$ (5)

where, *γ*_*th*_ is the threshold SNR required to ensure successful decoding at the relay.

Nearly capacity achieving codes (Non-ideal codes) such as repeat-accumulate code and low density parity check codes (LDPC) have a similar performance to that of the ideal code with identical code rate. However, the beauty of an ideal code is that it makes the analysis very simple, e.g., for a specific code rate, modulation and channel SNR, we can easily determine the status for successful decoding by observing (4) or (5).

So far, the link between the source and the *l*th relay is described. The next subsections explain different forwarding techniques at a relay.

## 4 Relaying techniques

Each relay can apply different forwarding techniques to the received signal from the source. In the literature these techniques are also termed as relaying protocols. These protocols are discussed with the assumption that perfect channel knowledge is available at each receiving node.

### 4.1 Amplify-and-forward (Non-regenerative relaying)

Amplify-and-forward (AF) is the simplest forwarding technique [1, 2], where, the relay does not decode the signal. It scales the received signal and transmits it to the destination. The advantage of this scheme is the reduced hardware complexity. However, as a drawback, the useful signal as well as the noise are amplified. The signal forwarded from the *l*th relay can be represented as

$$\begin{array}{cc} & x_{l}=\sqrt{\alpha_{l}}\cdot G_{l}\cdot y_{l}\;\text{with,}\;G_{l}=\frac{1}{\sqrt[{}]{\alpha_{s}{\left|h_{ls}\right|}^{2}+\sigma_{n}^{2}}} \end{array}$$ (6)

The received signal **y**_*l*_, is scaled with the factor *G*_*l*_ to limit the average transmit power to *α*_*l*_.

From (3), the respective SNR at the destination from the *l*th relay can be given as [8]

$$\begin{array}{ccc} \gamma_{l}^{AF} & = & \frac{\alpha_{s}\cdot \gamma_{ls}\cdot \alpha_{l}\cdot \gamma_{dl}}{\alpha_{s}\cdot \gamma_{ls}+\alpha_{l}\cdot \gamma_{dl}+1}. \end{array}$$ (7)

The end-to-end SNR delivered by all the *L* AF relays and source after maximum ratio combining (MRC) is

$$\begin{array}{c} \gamma_{MRC}^{AF}=\alpha_{s}\cdot \gamma_{ds}+\sum_{l=1}^{L}\gamma_{l}^{AF}, \end{array}$$ (8)

where, *α*_*s*_ ⋅ *γ*_*ds*_ is the signal to noise ratio of the signal at the destination (**y**_*ds*_) through the direct link.

### 4.2 Decode-and-forward (Regenaritive relaying)

In the case of decode-and-forward, the relay tries to decode the received signal. In the case of successful decoding, it re-encodes the signal **u** using identical code as used by the source. Afterwards, the modulated sequence **x**_*s*_ is generated. If there is decoding failure the relay remains silent, otherwise, errors will be propagated. Only relays with successful decoding are candidates for transmission. Thus, the *l*th relay transmits the signal

$$\begin{array}{c} x_{l}=\sqrt{\alpha_{l}}\cdot x_{s}. \end{array}$$ (9)

The end-to-end SNR delivered by all the *L* AF relays and the source after maximum ratio combining (MRC) is

$$\begin{array}{c} \gamma_{MRC}^{DF}=\alpha_{s}\gamma_{ds}+\sum_{l=1}^{L}\alpha_{l}\gamma_{dl}. \end{array}$$ (10)

### 4.3 Amplify-or-decode & forward (Hybrid relaying)

As stated in the introduction, AF and DF have inherent problems of noise amplification and error propagation. The alternative approach, the Adaptive Amplify-or-Decode & Forward (ADF) was presented in [4]. In this technique, the relay is not fixed to a specific forwarding scheme, the relay adapt itself according to the quality of the received signal. This the reason it is named adaptive scheme. For ADF, the relay forwards the re-encoded signal if it is able to successfully decode the received signal. Contrary, it applies amplify-and-forward in the case of a decoding failure. Thus, the signal transmitted from the *l*th relay is

$$\begin{array}{c} x_{l}=\{\begin{array}{cc} \sqrt[{}]{\alpha_{l}}\cdot x_{s} & \text{for}\;\alpha_{s}\cdot \gamma_{ls}\geq \gamma_{th}\\ \sqrt[{}]{\alpha_{l}}\cdot \frac{y_{l}}{\sqrt[{}]{\alpha_{s}\cdot {\left|h_{ls}\right|}^{2}+\sigma_{n}^{2}}} & \text{for}\;\alpha_{s}\cdot \gamma_{ls}<\gamma_{th} \end{array}\;, \end{array}$$ (11)

where, *α*_*s*_ ⋅ *γ*_*ls*_ is the received SNR via the source-relay link and *γ*_*th*_ is the threshold SNR to ensure successful decoding.

For ADF according to (11) the received SNR at the destination via *l*th relay can be written as

$$\begin{array}{c} \gamma_{l}^{ADF}=\{\begin{array}{cc} \alpha_{l}\cdot \gamma_{dl} & \text{for}\;\alpha_{s}\cdot \gamma_{ls}\geq \gamma_{th}\\ \gamma_{l}^{AF} & \text{for}\;\alpha_{s}\cdot \gamma_{ls}<\gamma_{th} \end{array}\;. \end{array}$$ (12)

Finally, the entire SNR at the destination after maximum ratio combining can be given as

$$\begin{array}{c} \gamma^{ADF}=\alpha_{s}\cdot \gamma_{ds}+\sum_{l=1}^{L}\gamma_{l}^{ADF}, \end{array}$$ (13)

### 4.4 Simulation results

**Fig 3:**
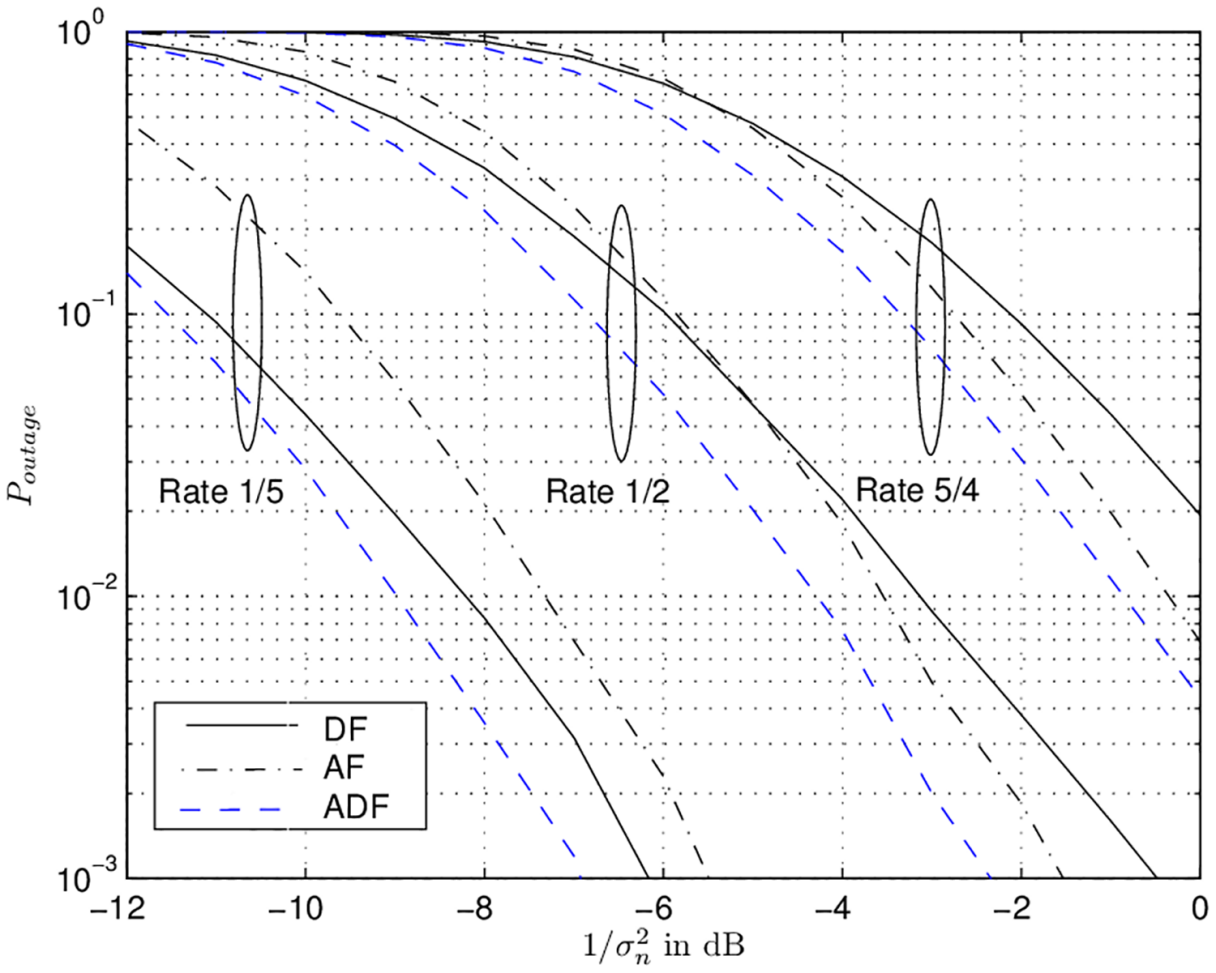
Outage probability vs. the pseudo SNR $\frac{1}{\sigma_{n}^{2}}$ for amplify-and-forward, decode-and-forward, and adaptive amplify-or-decode & forward.

This subsection presents the outage probability curves for the relaying protocols described so far in this section. These simulations were performed by using an ideal code with BPSK modulation and *L* = 4 relays. In Fig 3, the outage probability curves vs. the pseudo SNR $1/\sigma_{n}^{2}$ for different effective rates *R* = [1/5, 1/2, 4/5] are shown. The source and destination are placed at normalized distance of *d*_*ds*_ = 1 and *α*_*s*_ = *α*_*l*_ = 1 ∀*l* holds. The relays are uniformly distbuted on a vertical line of normalized length 0.4 in the middle of the source and relays. It is clear that adaptive amplify-or-decode & forward (ADF) outperforms amplify-and-forward (AF) and decode-and-forward (DF) by 1 to 2 dB for the given rates. At low rate *R* = 1/5 DF delivers a gain of 0.8 dB over AF in the high SNR regime. This is due to the reason that at this low rate almost all of the relays can decode successfully and transmit error free signals to the destination. Contrary, AF forwards a signal corrupted with noise, consequently, leading to high outage rate at the destination. It can also be observed that AF performs better than DF in the high SNR regime for *R* = 1/2 and *R* = 5/4. That is logical because in the case of DF most of the relays can not decode successfully at these high rates and remain silent. The relays with AF do not remain silent, they forward the noisy signals with high SNR. This leads to low outage.

**Fig 4:**
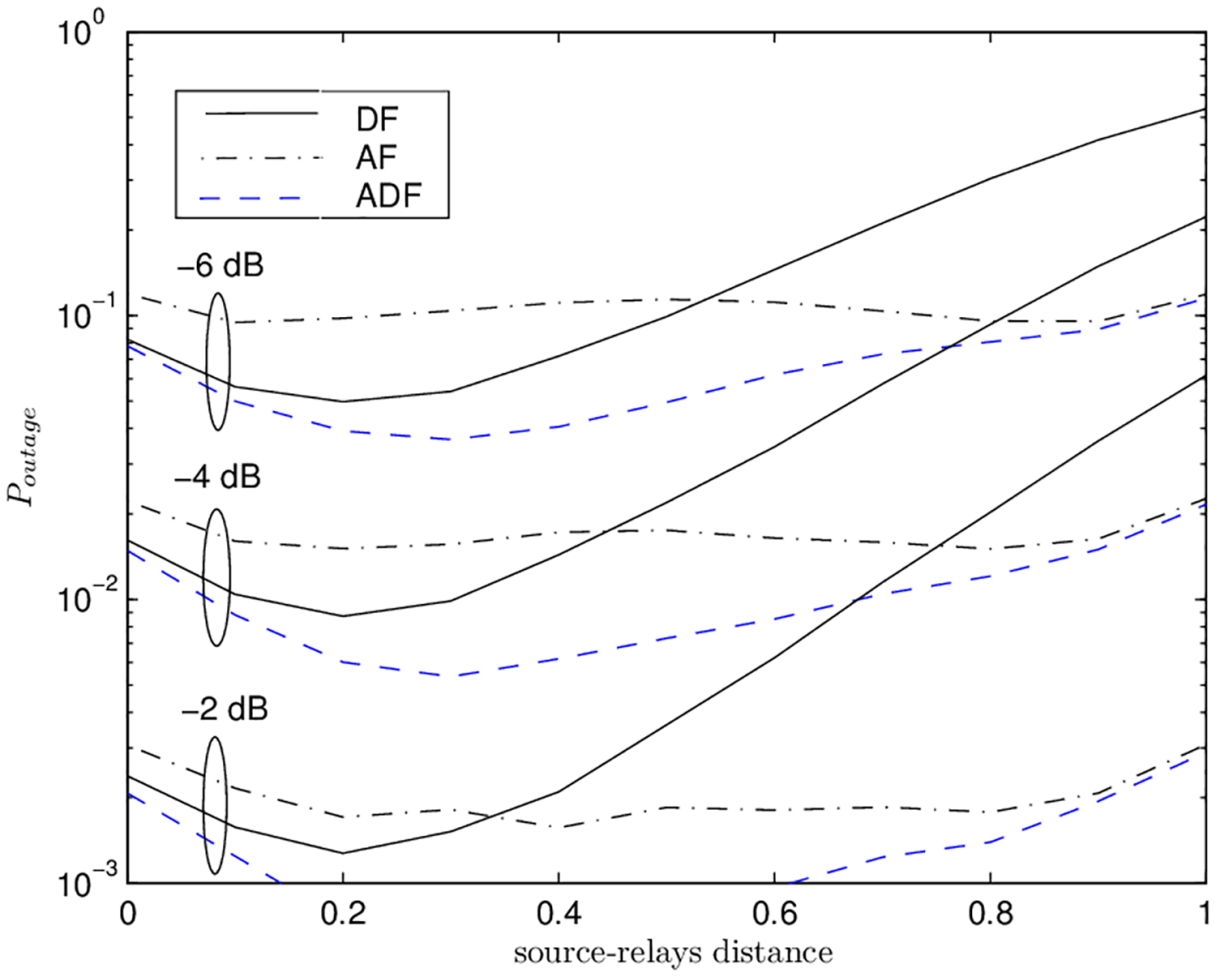
Outage probability vs. source-relays distance for amplify-and-forward, decode-and-forward, and adaptive amplify-or-decode & forward with different values of pseudo SNRs $\frac{1}{\sigma_{n}^{2}}$.

Fig 4 depicts the outage probability curves with pseudo SNR $1/\sigma_{n}^{2}$ -6 dB, -4 dB, and -2 dB vs. the source-relays distance for effective rate *R* = 1/2. The significant gains provided by ADF over AF and DF are eminent. The performance of DF improves initially as the relays move towards the middle. However, it degrades later as the relays move away from 0.2 towards the destination. This is due to the reason that initially most of the relays can decode successfully and as they move the relays-destination path-loss decreases. From 0.2 onwards, most of the relays cannot decode successfully due to increased source-relay path-loss. This causes high outage.

## 5 Power allocation with ideal codes and perfect channel knowledge

In this section, the problem formulation for the power optimization and the respective solution for adaptive ADF relaying protocols are summarized. For the optimization, a central node with perfect channel knowledge for each link is assumed. The central node delivers the optimized power values to the respective nodes. An error free signaling channel is assumed.

The aim of the optimization is to ensure successful decoding at the destination by consuming the minimum possible power. Mathematically, the optimized power set for the source and the *L* relays is given as

$$\begin{array}{cc} & \alpha_{set}^{*}=\underset{\alpha_{set}}{\text{argmin}}[\alpha_{s}+\sum_{l=1}^{L}\alpha_{l}] \end{array}$$ (14)

such that,

$$\begin{array}{cc} & \alpha_{s}\cdot \gamma_{ds}+\sum_{l=1}^{L}\gamma_{l}^{ADF}\geq \gamma_{th}\;, \end{array}$$ (15)

$$\begin{array}{cc} & 0<\alpha_{s}\leq \alpha_{max}, \end{array}$$ (16)

$$\begin{array}{cc} & 0\leq \alpha_{l}\leq \alpha_{max},\;\text{for}\;l\in \left\{1,2,.....L\right\}.\;\;\;\;\; \end{array}$$ (17)

Here, *α*_*set*_ = [*α*_*s*_, *α*_1_, *α*_2_, ….*α*_*L*_] holds. If the source and all the available relays are activated with maximum power (*α*_*max*_) and constraint (15) is not fulfilled, then an outage is declared.

For these sort of problems, one can use heuristic algorithms such as [21, 22] as done in [23] or one can use optimal solution using Lagrangian method. The heuristic algorithm may deliver fast solution but this may not be optimal. An optimal Lagrangian based solution to this problem was presented in [8]. Due to the space limitation only the principle is provided in this paper. Interested readers can see the solution in detail in the aforementioned reference. The authors used the Lagrangian method to solve the power allocation problem (14). The increase in the source power can make some relays decode successfully, this introduces a step function in (15). Therefore, an optimal solution is determined by keeping the source power constant. Optimizations are performed for different values of the source power and the one which consumes the minimum sum power is selected as the optimum. For a fixed source power, the objective function as well as the constraint functions are convex w.r.t. *α*_*l*_, therefore, convex optimization with Lagrangian multipliers is used. It is assumed that the relays with indices 1 ≤ *l* ≤ *L*_*AF*_ use amplify-and-forward, while, the relays with *L*_*AF*_ + 1 ≤ *l* ≤ *L* use decode-and-forward (they decoded successfully). Consequently, the respective Lagrangian function with Lagrangian multipliers λ, *μ*_*l*_, *μ*_*s*_ for (14) can be written as

$$\begin{array}{cc} L & =(\alpha_{s}+\sum_{l=1}^{L}\alpha_{l})+\lambda (-\alpha_{s}\cdot \gamma_{ds}-\sum_{l=1}^{L_{AF}}\gamma_{l}^{AF}-\;\;\;\;\;\sum_{l=L_{AF}+1}^{L}\;\;\;\;\;\alpha_{l}\cdot \gamma_{dl}+\gamma_{th})\\ & -\mu_{s}\cdot \alpha_{s}+\mu_{s}^{\prime }\left(\alpha_{s}-\alpha_{max}\right)-\sum_{l=1}^{L}\mu_{l}\cdot \alpha_{l}+\sum_{l=1}^{L}\mu_{l}^{\prime }\cdot \left(\alpha_{l}-\alpha_{max}\right). \end{array}$$ (18)

After taking the derivatives of this function w.r.t. λ and *α*_*l*_ and through complementary slackness conditions, the solution for the relays with AF and variable λ is

$$\begin{array}{l} \alpha_{l}^{*}={\left[\frac{-b+\sqrt{b^{2}-4ac}}{2a}\right]}_{0}^{\alpha_{max}}\\ \;\text{for}\;l\in \left\{1,2,3,..L_{AF}\right\}\\ \text{where,}\\ a=\gamma_{dr_{l}}^{2},\\ b=2\gamma_{dr_{l}}\left(1+\gamma_{r_{l}s}\alpha_{s}\right),\\ c={\left(1+\alpha_{s}\gamma_{r_{l}s}\right)}^{2}-\lambda \left(1+\alpha_{s}\gamma_{r_{l}s}\right)\left(\alpha_{s}\gamma_{dr_{l}}\gamma_{r_{l}s}\right). \end{array}$$ (19)

Furthermore in (19), $z={\left[x\right]}_{0}^{y}$ means that if *x* > *y* then *z* = *y* else if *x* < 0 then *z* = 0 holds. Otherwise, *z* = *x* holds. The problem is further simplified by temperory considering only a single best (having the best relay-destination SNR) relay among decode-and-forward relays, the index of this relay is assumed to be $\hat{l}$. Then, we have

$$\begin{array}{c} \alpha_{\hat{l}}=1/\gamma_{dr_{\hat{l}}}(\gamma_{th}-\alpha_{s}\gamma_{ds}-\sum_{l=1}^{L_{AF}}\frac{\alpha_{s}\gamma_{r_{l}s}\alpha_{l}^{*}\gamma_{dr_{l}}}{\alpha_{s}\gamma_{r_{l}s}+\alpha_{l}^{*}\gamma_{dr_{l}}+1}). \end{array}$$ (20)

if $\alpha_{\hat{l}}\leq \alpha_{max}$ holds then the solution for this relay with variable λ is $\alpha_{\hat{l}}^{*}={\left[\alpha_{\hat{l}}\right]}_{0}^{\alpha_{max}}$.

In [8], an optimal value for variable λ is found through bisection search such that (15) is satisfied. This algorithm with a single DF relays is extended to multiple DF relays by considering each DF relay one by one and removing the one whose power is already optimized.

## 6 ARQ strategies

Sec. 5 presented the power optimization for ideal codes with perfect channel knowledge. The next subsection 6.1 explains a strategy in which a central node allocates power based on imperfect channel knowledge using the power optimization of Sec. 5. This optimization algorithm assumed perfect channel knowledge. Therefore, this strategy provides sub-optimum power allocation. For this strategy, the impact of channel uncertainty on the power allocation is discussed in the simulation results.

### 6.1 ARQ strategy with imperfect CSI

In practice, obtaining the channel state information at a relay or at the destination is an estimation problem and can generally not be error free. The relays and destination deliver this imperfect CSI to the central node only once in the beginning. The goal of the central node is to ensure successful decoding at the destination by allocating sufficient power to the nodes such that the recieved SNR at the destination is equal to the threshold SNR *γ*_*th*_. However, due to the channel uncertainty, it can only estimate the received SNR (44) instead of the actual SNR. Therefore, it allocates the power to all of the nodes such that the estimated SNR is greater than or equal to *γ*_*th*_. It may happen that the estimated SNR at the central node is greater than the true experienced SNR (43) at the destination, thus, the cooperation from the nodes does not lead to successful decoding. In the case of unsuccessful decoding, the destination/relays feedback the status of (un)successful decoding to the central node, and, power optimization at the central node is repeated. Multiple optimizations are performed until the destination decodes successfully or the maximum power limit for each node in (14) is reached. During each repetition, the same number of bits are transmitted, thus, the total transmit power is directly proportional to the consumed energy. When the power limit is reached outage is declared. For each subsequently repeated optimization, the central node must increment the threshold SNR *γ*_*th*_ to increase the power and consequently results in more received SNR at the destination. Each repeated optimization is followed by retransmissions (repetitions) from the nodes with non-zero assigned power. Here, *q* denotes the total number of repetitions (including the first one from the source) during all of the repeated optimizations. Furthermore, let, *j* − 1 represents the number of recurrent optimizations and $\gamma_{th}^{\left(j\right)}$ is the incremented SNR for the *j*th re-optimization. Moreover, $\alpha_{s}^{\left(j\right)}$, and $\alpha_{l}^{\left(j\right)}$ denote the optimum power calculated for the source and the *l*th relay respectively to achieve SNR $\gamma_{th}^{\left(j\right)}$ at the destination. Then, the power allocated to the source and *l*th relay during the *j*th recurrent optimization is the difference between the optimized power in *j*th optimization and *j* − 1th optimization, i.e.,

$$\begin{array}{c} \Delta \alpha_{s}^{\left(j\right)}=\alpha_{s}^{\left(j\right)}-\alpha_{s}^{\left(j-1\right)}\;\text{and}\; \end{array}$$ (21)

$$\begin{array}{c} \Delta \alpha_{l}^{\left(j\right)}=\alpha_{l}^{\left(j\right)}-\alpha_{l}^{\left(j-1\right)} \end{array}$$ (22)

respectively. Moreover, $\gamma_{th}^{\left(1\right)}=\gamma_{th}$ holds, and the threshold SNR for *j* > 1 is incremented as below

$$\begin{array}{c} \gamma_{th}^{\left(j\right)}=\gamma_{th}^{\left(j-1\right)}+\left|\gamma_{th}-{\hat{\gamma }}^{\left(j-1\right)}\right|+\omega \cdot \gamma_{th} \end{array}$$ (23)

Here, $|\gamma_{th}-{\hat{\gamma }}^{\left(j-1\right)}|$ is the difference between the threshold SNR required at the destination and the estimated experienced SNR (44) during the *j* − 1th optimization. Furthermore, if *ω* = 0 holds, and $\gamma_{th}\to {\hat{\gamma }}^{\left(j-1\right)}$, then, it implies that $\gamma_{th}^{\left(j\right)}\to \gamma_{th}^{\left(j-1\right)}$. This results in the same optimized power values for the *j*th optimization as for *j* − 1 i.e., $\alpha_{l}^{\left(j\right)}\to \alpha_{l}^{\left(j-1\right)}\Rightarrow \alpha_{l}^{\left(j\right)}\to 0$ holds. This leads to extremely slow convergence for the ARQ process to ensure successful decoding at the destination. Therefore, *ω* ⋅ *γ*_*th*_ is added to speed up the ARQ process and reach successful decoding quickly. The impact of *ω* on the performance of the system is demonstrated in the simulation results.

## 7 SNR with imperfect channel knowledge & ideal coding

In this section, the end-to-end SNR for a relay network with imperfect channel knowledge and ideal coding in conjunction with ARQ is calculated. It is assumed that the central node, relays as well as the destination do not have perfect channel state information (CSI). Each relay and the destination estimate the source-relay and relay-destination channel respectively. In the beginning, the channel estimates from all the nodes are forwarded over an error free signaling channel to the central node for power allocation. We follow the MMSE channel estimation model presented in [10]. The estimate has the following relation with the true channel and the estimation error represented by *e* (without subscript).

$$\begin{array}{c} h=\hat{h}+e. \end{array}$$ (24)

The estimate as well as the estimation error are considered to be complex Gaussian distributed with mean zero and variance given by $\sigma_{\hat{h}}^{2}=\sigma_{h}^{2}-\sigma_{e}^{2}$ and $\sigma_{e}^{2}=\frac{\sigma_{h}^{2}\cdot \sigma_{n}^{2}}{\sigma_{n}^{2}+\sigma_{h}^{2}\cdot N_{p}}$ respectively. The variables $\sigma_{h}^{2}$ and *N*_*p*_ deonote the variance of the true channel and the number of pilot symbols used for the estimation respectively. The central node allocates power to the nodes based on the imperfect channel information, this is followed by transmissions from the source and relays. Due to the channel uncertainty, the allocated power may not suffice to achieve successful decoding at the destination (See Sec. 6.1 for details). Therefore, an ARQ process is started, where, multiple power optimizations are done, each optimization is followed by transmissions from the nodes. Thus, each node may transmit more than once with a newly optimized power. During each retransmission from the source and relays the channel estimation is done and relays or destination update the channel state information (CSI). In order to have low signalling overhead, the central node receives the CSI only in the beginning and the updated CSI is not delivered to the it after every retransmission. Let, *q* denotes the number of transmissions received at the destination from all the nodes during multiple optimizations of each ARQ process. Moreover, *l*_*q*_ represents the index of a relay, which repeats during the *q*th transmission. The channel estimate from the source to the *l*_*q*_th relay and to the destination is denoted by ${\hat{h}}_{l_{q}s}$ and ${\hat{h}}_{ds_{q}}$ respectively. The channel estimate from the *l*_*q*_th relay to the destination is given by ${\hat{h}}_{dl_{q}}$.

Furthermore, $Q_{s}^{q}\subset \left\{1,2,3,4...q-1\right\}$ is the set of the number of transmissions when the source was selected to repeat. The signal received at the *l*_*q*_th relay from the source can be written as

$$\begin{array}{cc} y_{l_{q}s} & =h_{l_{q}s}\cdot \sqrt[{}]{\alpha_{s_{q}}}\cdot x_{s}+n_{l_{q}s}, \end{array}$$ (25)

$$\begin{array}{cc} y_{l_{q}s} & =\left({\hat{h}}_{l_{q}s}+e_{l_{m}s}\right)\cdot \sqrt[{}]{\alpha_{s_{q}}}\cdot x_{s}+n_{l_{q}s}. \end{array}$$ (26)

By putting the value of *h*_*l*_*q*_*s*_ from (24) in the above equation, we get,

$$\begin{array}{cc} y_{l_{q}s} & =\underset{\text{signal}}{\underset{︸}{{\hat{h}}_{l_{q}s}\cdot \sqrt[{}]{\alpha_{s_{q}}}\cdot x_{s}}}+\underset{\text{entire}\;\text{noise}}{\underset{︸}{e_{l_{m}s}\cdot \sqrt[{}]{\alpha_{s_{q}}}\cdot x_{s}+n_{l_{q}s}}}. \end{array}$$ (27)

References [24] and [11] also consider the term “entire noise” in (27) as noise.

Each relay performs maximum ratio combining on the $\left|\right|Q_{s}^{q}\left|\right|$ received signals by scaling the received signal with $\sqrt{\alpha_{s_{q}}}{\hat{h}}_{l_{q}s}^{*}$. Afterwards, the addition of the signals result in the maximum ratio combining (MRC) and the respective output of the MRC is

$$\begin{array}{c} {\bar{y}}_{l_{q}s}=\sum_{m\in Q_{s}^{q}}\alpha_{s_{m}}{\left|{\hat{h}}_{l_{m}s}\right|}^{2}\cdot x_{s}+\sum_{m\in Q_{s}^{q}}\sqrt{\alpha_{s_{m}}}{\hat{h}}_{l_{m}s}^{*}(\sqrt{\alpha_{s_{m}}}\cdot e_{l_{m}s}\cdot x_{s}+n_{l_{m}s}). \end{array}$$ (28)

Then, similar to [25], the respective instantaneous SNR for this signal can be given as

$$\begin{array}{c} \gamma_{l_{q}s}=\frac{{\left|\sum_{m\in Q_{s}^{q}}\alpha_{s_{m}}{\left|{\hat{h}}_{l_{m}s}\right|}^{2}\right|}^{2}}{{\left|\sum_{m\in Q_{s}^{q}}\alpha_{s_{m}}\cdot e_{l_{m}s}\cdot {\hat{h}}_{l_{q}s}^{*}\right|}^{2}+\sum_{m\in Q_{s}^{q}}\alpha_{s_{m}}\cdot {\left|{\hat{h}}_{l_{q}s}^{*}\right|}^{2}\sigma_{n}^{2}}. \end{array}$$ (29)

As adaptive ADF relaying protocol is considered, in which, the relay forwards the re-encoded signal if it is able to successfully decode the received signal. Otherwise, in the case of a decoding failure, it applies amplify-and-forward. Thus, the signal transmitted from the *l*_*q*_th relay is

$$\begin{array}{c} x_{l_{q}}=\{\begin{array}{cc} \sqrt[{}]{\alpha_{l_{q}}}\cdot x_{s} & \text{for}\;\gamma_{l_{q}s}\geq \gamma_{th}\\ \sqrt[{}]{\alpha_{l_{q}}}\cdot {\bar{y}}_{l_{q}s}\cdot \beta_{l_{q}} & \text{for}\;\gamma_{l_{q}s}<\gamma_{th} \end{array}\;, \end{array}$$ (30)

where, $\beta_{l_{q}}=\frac{1}{\sqrt{{\left(\sum_{m\in Q_{s}^{q}}\alpha_{s_{m}}{\left|{\hat{h}}_{l_{q}s}\right|}^{2}\right)}^{2}+\sum_{m\in Q_{s}^{q}}\alpha_{s_{m}}\cdot {\left|{\hat{h}}_{l_{q}s}\right|}^{2}\cdot \sigma_{n}^{2}}}$ is a normalization factor such that the average transmitted power for the *q*th retransmission is *α*_*l*_*q*__.

Similarly, at the destination, the equivalent received baseband signal from the *l*th relay can be written as

$$\begin{array}{cc} y_{l_{q}}= & \underset{\text{signal}}{\underset{︸}{{\hat{h}}_{dl_{q}}x_{l_{q}}}}+\underset{\text{entire}\;\text{noise}}{\underset{︸}{e_{dl_{q}}x_{l_{q}}+n_{dl_{q}}}}. \end{array}$$

The destination performs maximum ratio combining over all the received signals from the source and the relays to decode the source’s transmitted signal.

In this section, the end-to-end (E2E) performance for a relay network presented in Sec. 2 under channel estimation errors is derived. The signal received at the destination during the *q*th transmission from the *l*_*q*_th relay with AF can be represented as

$$\begin{array}{c} y_{l_{q}}=h_{dl_{q}}\cdot \sqrt[{}]{\alpha_{l_{q}}}\cdot {\bar{y}}_{l_{q}s}\cdot \beta_{l_{q}}+n_{dl_{q}}. \end{array}$$ (31)

This can also be expressed as

$$\begin{array}{ll} y_{l_{q}}=h_{dl_{q}}\cdot \sqrt{\alpha_{l_{q}}}\left(\sum_{m\in Q_{s}^{q}}\alpha_{s_{m}}{\left|{\hat{h}}_{l_{m}s}\right|}^{2}\cdot x_{s}+\sum_{m\in Q_{s}^{q}}\sqrt{\alpha_{s_{m}}}{\hat{h}}_{l_{m}s}^{*}\left(\sqrt{\alpha_{s_{m}}}\cdot e_{l_{m}s}\cdot x_{s}+n_{l_{m}s}\right)\right)\times & \left(32\right)\\ \beta_{l_{q}}+n_{dl_{q}}. & \left(33\right) \end{array}$$

After the substitution of ${\hat{h}}_{dl_{q}}+e_{dl_{q}}$ for *h*_*dl*_*q*__ and with simple mathematical manipulation, we obtain,

$$\begin{array}{cc} y_{l_{q}}= & \underset{\text{signal}}{\underset{︸}{\sqrt[{}]{\alpha_{l_{q}}}{\hat{h}}_{dl_{q}}\sum_{m\in Q_{s}^{q}}\alpha_{s_{m}}{\left|{\hat{h}}_{l_{m}s}\right|}^{2}x_{s}\beta_{l_{q}}}}+\sqrt[{}]{\alpha_{l_{q}}}({\hat{h}}_{dl_{q}}+e_{dl_{q}})\sum_{m\in Q_{s}^{q}}\sqrt{\alpha_{s_{m}}}{\hat{h}}_{l_{m}s}^{*}n_{l_{m}s}\beta_{l_{q}}+\\ & \sqrt[{}]{\alpha_{l_{q}}}\cdot x_{s}\cdot \beta_{l_{q}}\underset{\xi_{q}}{\underset{︸}{\sum_{m\in Q_{s}^{q}}\alpha_{s_{m}}((|{\hat{h}}_{l_{m}s}{|}^{2}+{\hat{h}}_{l_{m}s}^{*}e_{l_{m}s})e_{dl_{q}}+{\hat{h}}_{dl_{q}}{\hat{h}}_{l_{m}s}^{*}e_{l_{m}s})}}+n_{dl_{q}} \end{array}$$ (34)

In (34), the part “signal” is the useful signal, while, the summation of the remaining terms is the entire noise [11, 24]. Similarly, when the relay uses decode-and-forward, the received signal at the destination from the *l*_*q*_th relay/source (for source *l* = *s* holds) can be given as

$$\begin{array}{cc} y_{l_{q}}= & \underset{\text{signal}}{\underset{︸}{\sqrt[{}]{\alpha_{l_{q}}}{\hat{h}}_{dl_{q}}x_{s}}}+\underset{\text{entire}\;\text{noise}}{\underset{︸}{\sqrt[{}]{\alpha_{l_{q}}}e_{dl_{q}}x_{s}+n_{dl_{q}}}}. \end{array}$$ (35)

Let *Q* is the total number of transmissions received at the destination from the source and from all the relays at the end of the current repeated optimization. In order to calculate the SNR at the destination, the destination should combine the *Q* received signals in an optimal way. It can be seen from (34) and (35) that the noise terms are correlated due to the repetition of the same noise samples (**n**_*l*_*m*_*s*_) from the same relay. Therefore, the conventional maximum ratio combiner is no more optimal. In [26], it is shown that in this case the optimal matched filter is given by

$$\begin{array}{c} w=\Phi^{-1}h^{Eff}. \end{array}$$ (36)

Here, Φ is the *Q* × *Q* covariance matrix between the received noise sequences. The *q*th element of the effective channel (being *Q* × 1 column vector) can be given as

$$h_{q}^{Eff}=\{\begin{array}{cc} \sum_{m\in Q_{s}^{q}}\alpha_{s_{m}}{\left|{\hat{h}}_{l_{m}s}\right|}^{2}\beta_{l_{q}}\sqrt{\alpha_{l_{q}}}\cdot {\hat{h}}_{dl_{q}} & \text{for}\;\gamma_{l_{q}s}<\gamma_{th}\\ \sqrt{\alpha_{l_{q}}}\cdot {\hat{h}}_{dl_{q}}\;\;\; & \text{for}\;\gamma_{l_{q}s}\geq \gamma_{th} \end{array}$$ (37)

Let, if the channel estimation errors are known (it is not possible in practice), then each element of Φ on row *q* and column *q*′ can be calculated as

$$\begin{array}{c} \Phi_{q,q^{\prime }}=\{\begin{array}{cc} S_{1}\; & \text{for}\;q=q^{\prime },\;\gamma_{l_{q}s}<\gamma_{th}\\ S_{2}\; & \text{for}\;q\neq q^{\prime },\;l_{q}=l_{q^{\prime }},\;\gamma_{l_{q}s}<\gamma_{th},\;\gamma_{l_{q^{\prime }}s}<\gamma_{th}\\ S_{3}\; & \text{for}\;q\neq q^{\prime },\;l_{q}\neq l_{q^{\prime }},\;\gamma_{l_{q}s}<\gamma_{th},\;\gamma_{l_{q^{\prime }}s}<\gamma_{th}\\ S_{4}\; & \text{for}\;q=q^{\prime },\;\gamma_{l_{q}s}\geq \gamma_{th}\\ S_{5}\; & \text{for}\;q\neq q^{\prime },\;\gamma_{l_{q}s}\geq \gamma_{th},\;\gamma_{l_{q^{\prime }}s}<\gamma_{th}\\ S_{6}\; & \text{for}\;q\neq q^{\prime },\;\gamma_{l_{q}s}<\gamma_{th},\;\gamma_{l_{q^{\prime }}s}\geq \gamma_{th}, \end{array} \end{array}$$

Here, *S*_1_ corresponds to the diagonal elements of the covariance matrix, i.e., covariance between two identical noise signals, where, the relay does not decode successfully. *S*_2_ is associated with the case when the two signals are different but they are transmitted from the same relay (*l*_*q*_) with unsuccessful decoding. *S*_3_ belongs to the case when the signal are transmitted from two different relays and both of the relays do not decode successfully. *S*_4_ corresponds to the diagonal elements of the covariance matrix when the relay decodes successfully. *S*_5_ and *S*_6_ indicates the covariance between two different signals where at least one relay decodes successfully. Moreovere, *S*_1_, …, *S*_6_ are

$$\begin{array}{cc} S_{1}= & \alpha_{l_{q}}\beta_{l_{q}}^{2}\left(\right|\xi_{q}{|}^{2}+|{\hat{h}}_{dl_{q}}+e_{dl_{q}}{|}^{2}\sum_{m\in Q_{s}^{q^{\prime }}}|{\hat{h}}_{l_{m}s}{|}^{2}\alpha_{s_{m}}\sigma_{n}^{2})+\sigma_{n}^{2},\\ S_{2}= & \sqrt{\alpha_{l_{q}}}\beta_{l_{q}}\xi_{q}\sqrt{\alpha_{l_{q^{\prime }}}}\beta_{l_{q^{\prime }}}\xi_{q^{\prime }}^{H}+\sqrt{\alpha_{l_{q}}}\sqrt{\alpha_{l_{q^{\prime }}}}\sum_{m\in Q_{s}^{q}\cap Q_{s}^{q^{\prime }}}\alpha_{s_{m}}|{\hat{h}}_{l_{m}s}{|}^{2}\sigma_{n}^{2}{\left|{\hat{h}}_{dl_{q}}+e_{dl_{q}}\right|}^{2}\beta_{l_{q}}\beta_{l_{q^{\prime }}},\\ S_{3}= & \sqrt{\alpha_{l_{q}}}\beta_{l_{q}}\xi_{q}\sqrt{\alpha_{l_{q^{\prime }}}}\beta_{l_{q^{\prime }}}\xi_{q^{\prime }}^{H},\;\;\;S_{4}=\alpha_{l_{q}}{\left|e_{dl_{q}}\right|}^{2}+\sigma_{n}^{2},\\ S_{5}= & \sqrt{\alpha_{l_{q}}}e_{dl_{q}}\sqrt{\alpha_{l_{q^{\prime }}}}\beta_{l_{q^{\prime }}}\xi_{q^{\prime }}^{H},\;\;\;S_{6}=\sqrt{\alpha_{l_{q^{\prime }}}}e_{dl_{q^{\prime }}}\sqrt{\alpha_{l_{q}}}\beta_{l_{q}}\xi_{q}^{H}. \end{array}$$

The exact values for the matrix Φ can not be calculated due to the reason that the channel estimation errors are not known, thus, the optimal matched filter **w** at the destination can not be calculated under the influence of estimation errors. Therefore, we only predict the optimal matched filter as

$$\begin{array}{c} \bar{w}={\bar{\Phi }}^{-1}h^{Eff}. \end{array}$$ (38)

The predicted covariance matrix $\bar{\Phi }$ is calculated from the optimal matched filter by averaging over all the estimation errors, i.e,

$$\begin{array}{c} \bar{\Phi }={\text{E}}_{e}\left[\Phi \right]. \end{array}$$ (39)

The element on the *q*th row and *q*′th column of this *Q* × *Q* matrix can be expressed as given below.

$$\begin{array}{c} {\bar{\Phi }}_{q,q^{\prime }}=\{\begin{array}{cc} {\bar{S}}_{1}\; & \text{for}\;q=q^{\prime },\;\gamma_{l_{q}s}<\gamma_{th}\\ {\bar{S}}_{2}\; & \text{for}\;q\neq q^{\prime },\;l_{q}=l_{q^{\prime }},\;\gamma_{l_{q}s}<\gamma_{th},\;\gamma_{l_{q^{\prime }}s}<\gamma_{th}\\ 0\; & \text{for}\;q\neq q^{\prime },\;l_{q}\neq l_{q^{\prime }},\;\gamma_{l_{q}s}<\gamma_{th},\;\gamma_{l_{q^{\prime }}s}<\gamma_{th}\\ {\bar{S}}_{4}\; & \text{for}\;q=q^{\prime },\;\gamma_{l_{q}s}\geq \gamma_{th}\\ 0\; & \text{for}\;q\neq q^{\prime },\;\gamma_{l_{q}s}\geq \gamma_{th},\;\gamma_{l_{q^{\prime }}s}<\gamma_{th}\\ 0\; & \text{for}\;q\neq q^{\prime },\;\gamma_{l_{q}s}<\gamma_{th},\;\gamma_{l_{q^{\prime }}s}\geq \gamma_{th} \end{array} \end{array}$$,

where,

$$\begin{array}{cc} {\bar{S}}_{1}= & \alpha_{l_{q}}\beta_{l_{q}}^{2}({\bar{\xi }}_{q}+(|{\hat{h}}_{dl_{q}}{|}^{2}+\sigma_{dl_{q}e}^{2})\sum_{m\in Q_{s}^{q}}\alpha_{s_{m}}{\left|{\hat{h}}_{l_{m}s}\right|}^{2}\sigma_{n}^{2})+\sigma_{n}^{2},\\ {\bar{S}}_{2}= & \sqrt{\alpha_{l_{q}}}\beta_{l_{q}}\sqrt{\alpha_{l_{q^{\prime }}}}\beta_{l_{q^{\prime }}}(|{\hat{h}}_{dl_{q}}{|}^{2}\sum_{m\in Q_{s}^{q}\cap Q_{s}^{q^{\prime }}}{\left|\alpha_{s_{m}}{\hat{h}}_{l_{m}s}\right|}^{2}\sigma_{l_{q}se}^{2})+\\ & \sqrt{\alpha_{l_{q}}\alpha_{l_{q^{\prime }}}}\sum_{m\in Q_{s}^{q}\cap Q_{s}^{q^{\prime }}}\alpha_{s_{m}}|{\hat{h}}_{l_{m}s}{|}^{2}\sigma_{n}^{2}\left(\right|{\hat{h}}_{dl_{q}}{|}^{2}+\sigma_{dl_{q}e}^{2})\beta_{l_{q}}\beta_{l_{q^{\prime }}}, \end{array}$$ (40)

$$\begin{array}{cc} {\bar{S}}_{4}= & \alpha_{l_{q}}\sigma_{dl_{q}e}^{2}+\sigma_{n}^{2}, \end{array}$$ (41)

$${\bar{\xi }}_{q}=\sum_{m\in Q_{s}^{q}}{\left|\alpha_{s_{m}}{\left|{\hat{h}}_{l_{m}s}\right|}^{2}\right|}^{2}\sigma_{dl_{q}e}^{2}+\sum_{m\in Q_{s}^{q}}{\left|\alpha_{s_{m}}{\hat{h}}_{l_{m}s}\right|}^{2}\sigma_{l_{q}se}^{2}\sigma_{dl_{q}e}^{2}+{\left|{\hat{h}}_{dl_{q}}\right|}^{2}\sum_{m\in Q_{s}^{q}}{\left|\alpha_{s_{m}}{\hat{h}}_{l_{m}s}\right|}^{2}\sigma_{l_{q}se}^{2}$$ (42)

and $\sigma_{dl_{q}e}^{2}=\text{E}[|e_{dl_{q}}{|}^{2}],\;\sigma_{l_{q}se}^{2}=\text{E}[|e_{l_{q}s}{|}^{2}]$ hold.

Finally, the true SNR and the predicted SNR can be given as

$$\begin{array}{cc} \gamma = & \frac{|{\bar{w}}^{H}h^{Eff}{|}^{2}}{|{\bar{w}}^{H}\Phi \bar{w}|}\;\text{and}\; \end{array}$$ (43)

$$\begin{array}{cc} \bar{\gamma }= & \frac{|{\bar{w}}^{H}h^{Eff}{|}^{2}}{|{\bar{w}}^{H}\bar{\Phi }\bar{w}|} \end{array}$$ (44)

respectively. The true SNR is actually the experienced SNR at the destination, therefore, it contains the term Φ instead of $\bar{\Phi }$ in the denominator.

## 8 Simulation results

This section deals with the simulation results explaining the impact of channel uncertainty on the power allocation, outage probability and the throughput in conjunction with ARQ. For all of these simulations, the source and destination are 1 meter apart. The relays are uniformly spaced on a vertical line of 0.4 meter exactly in the middle of the source and destination. Two relays are placed on the opposite ends of the line and the remaining two relays 0.2 meters apart from each of them.

### 8.1 Impact of channel uncertainty on ARQ relay network

**Fig 5:**
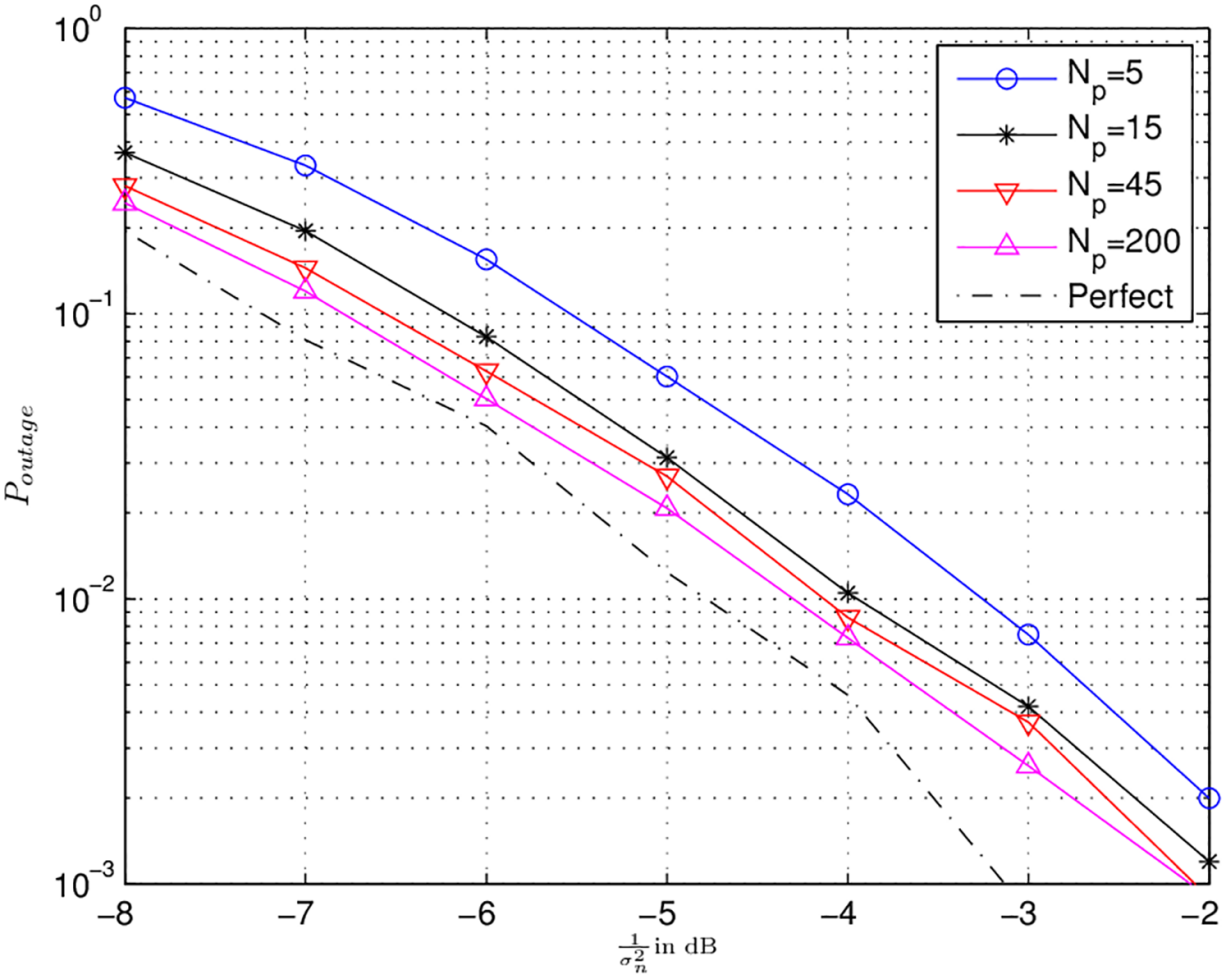
Outage probability curve for different number of estimation pilot symbols *N*_*p*_ are presented. The dash-dot-dash line represents the perfect CSI case.

**Fig 6:**
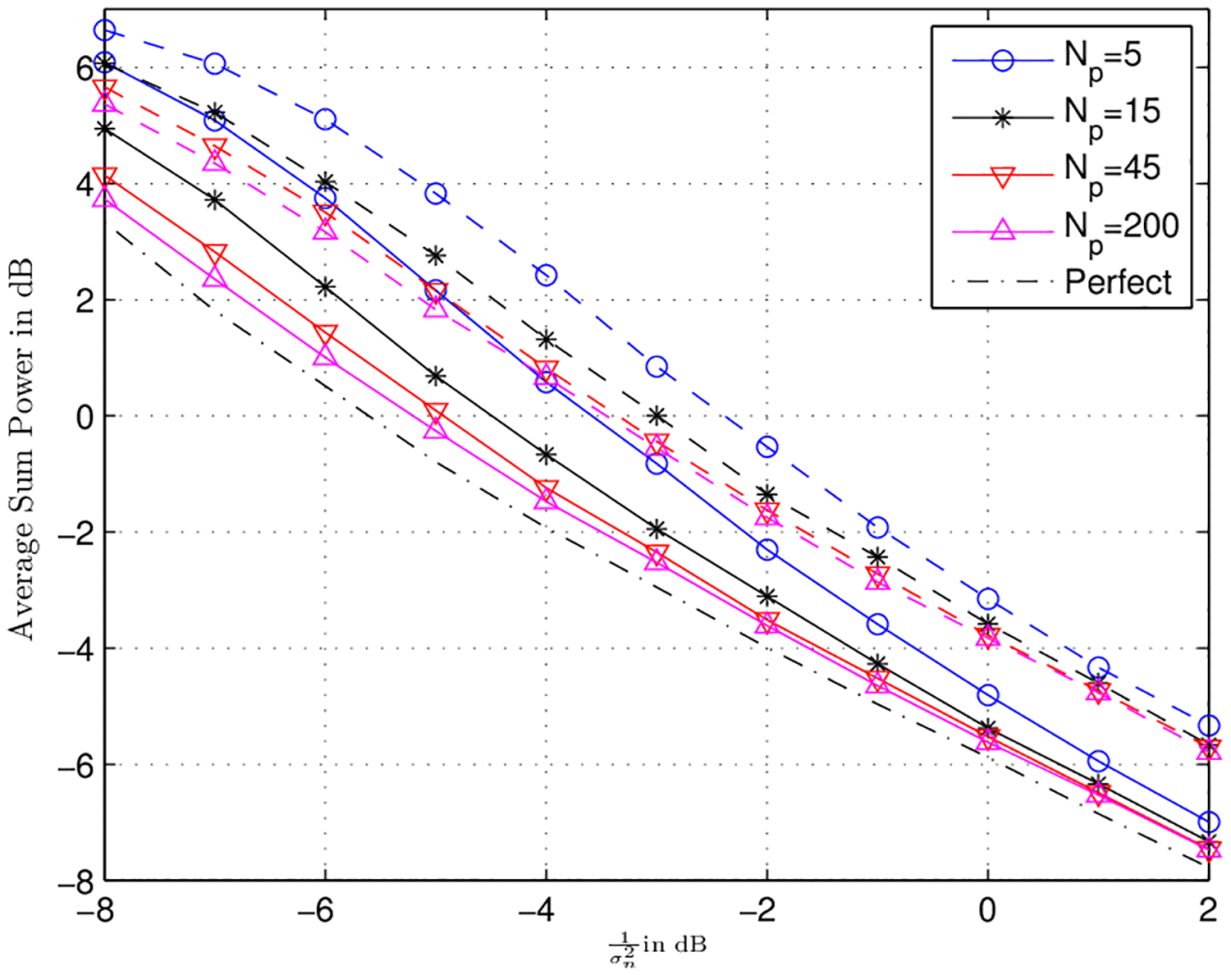
Average sum power consumed for different channel qualities with number of pilot symbols *N*_*p*_ are in dB depicted. The dash-dot-dash line represents the perfect CSI case.

**Fig 7:**
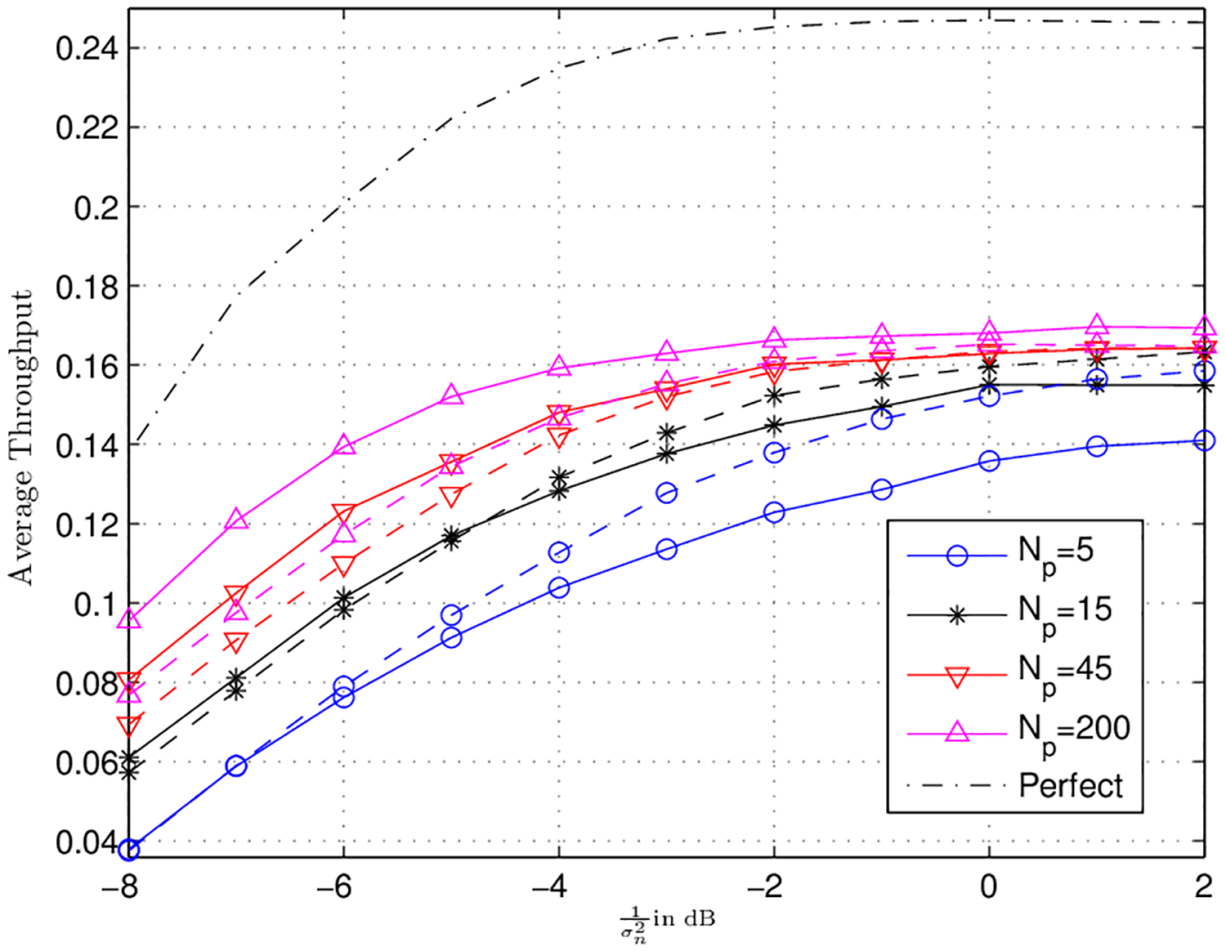
Average throughputs for different channel qualities with different number of pilot symbols *N*_*p*_ are shown. The dash-dot-dash line represents the perfect CSI case.

In this subsection, Figs 5 to 7 represent the simulation results for the ARQ relay network with channel uncertainty. For, the respective simulations, half rate ideal code, with BPSK modulation scheme was considered. BPSK is taken for the sake of simplicity, the same model can also be applied for higher order modulation. The effective code rate 0.5 leads to *γ*_*th*_ = 1.044. Moreover, 10000 channel realizations were simulated with *α*_*max*_ = 1.

Fig 5 shows the outage probability for different number of pilot symbols (*N*_*p*_) with *ω* equal to 0.001 and 1. Both the values of *ω* performs similarly, therefore, these curves represent both values. Here, the dash-dot-dash line represents the perfect channel knowledge case. The difference between perfect CSI and *N*_*p*_ = 5 is 1.5 at outage probability of 10^−3^. The same difference for *N*_*p*_ = [15, 45, 200] is 1 dB. This shows that even with small number of pilot symbols the outage probability for the power allocation strategy of Sec. 6.1 is not significantly deteriorated.

**Fig 8:**
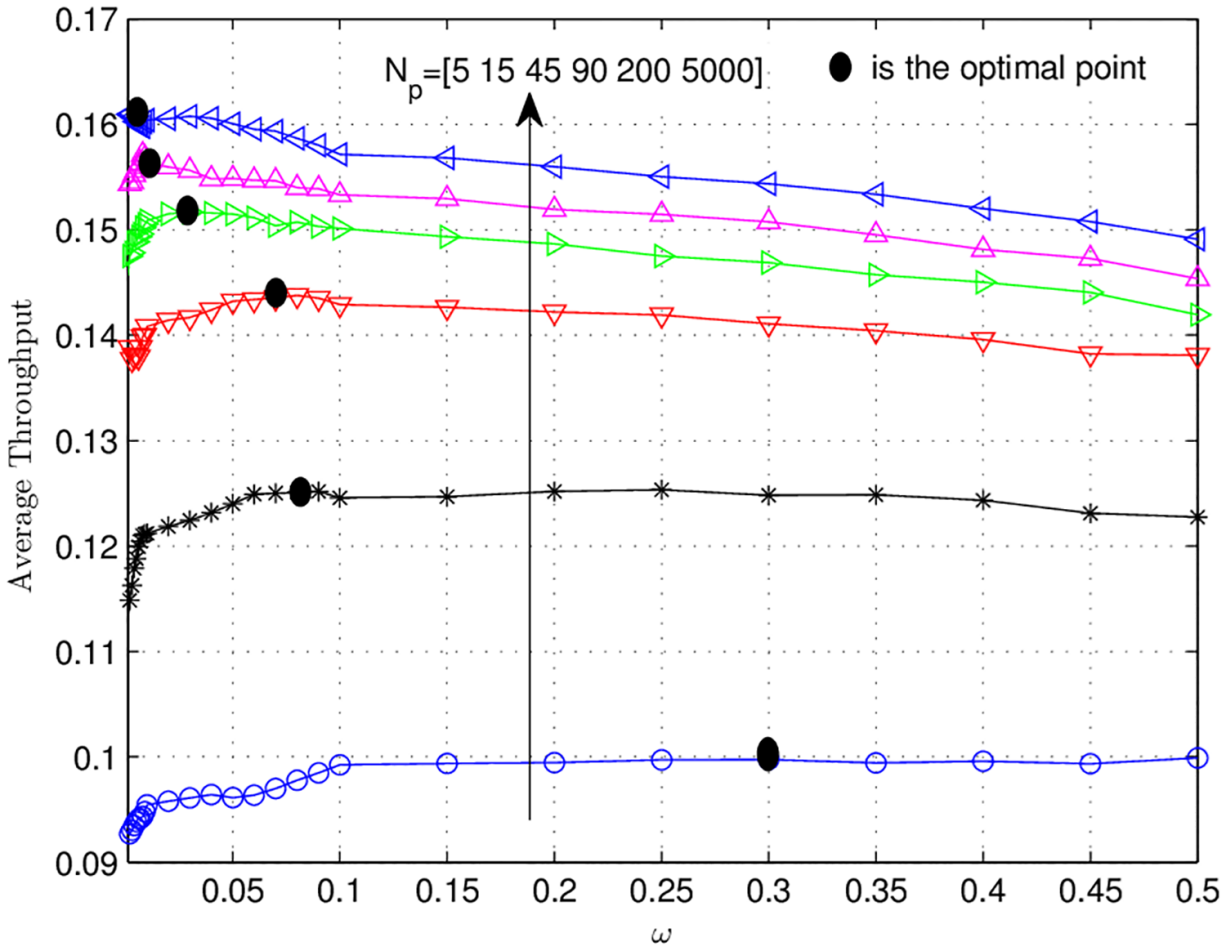
Average throughput curves vs. the trade-off factor *ω* are presented. The simulations for different number of pilot symbols *N*_*p*_ were performed.

In Fig 6, curves for the average sum power consumed in dB for different number of pilot symbols (*N*_*p*_ = [5, 15, 45, 200]) with *ω* equal to 0.001 (the solid lines) and 1 (the dashed lines) are depicted. For *ω* = 0.001, the difference between perfect CSI case and *N*_*p*_ = [5, 15, 45, 200] in the high SNR regime is roughly less than or equal to 0.5 dB. The same difference for *ω* = 1 is 1 dB. It demonstrates that regarding sum power consumption, the power allocation algorithm of Sec. 5 is very resielent to the channel imperfections. It also shows that higher value of *ω* requires more power as compared to the lower values. Finally, in Fig 7, the average throughput curves for each estimation quality with *ω* = 0.001 (the solid lines) and *ω* = 1 (dashed lines) are depicted. It can be seen that channel uncertainty has very strong impact on the throughput. The maximum throughput that can be achieved with perfect CSI is 0.25 b/s/Hz. This is due to the reason that the source-destination link has larger path-loss as compared to the source-relays links, therefore, most of the time the power optimization algorithm will activate the source and atleast one relay for transmissions. As, the effective code rate *R* is 0.5, thus, the maximum throughput that can be obtained is *R*/2 = 0.25 b/s/Hz. Moreover, it can be observed that the maximum throughput that can be obtained in the high SNR regime with imperfect CSI is 0.17 bits/s/Hz for *N*_*p*_ = 200 with *ω* = 0.001. This is 32% less than that of the perfect CSI case. For *N*_*p*_ = 5 and *ω* = 0.001, we have the lowest throughput equal to 0.12 bits/s/Hz. This is 52% less than the the maximum achievable throughput with perfect CSI. Furthermore, Fig 7 shows that *ω* = 0.001 achieve high throughput at *N*_*p*_ = 200 and low throughput at *N*_*p*_ = 5. This is due to the reason that at high value of *N*_*p*_, the impact of channel uncertainty is very small and less amount of extra power is needed to decode successfully at the destination during the re-optimization. It is known from Sec. 6.1 that for low value of *ω* the central node allocates less power, thus, less number of relays are activated to ensure successful decoding at the destination. This cause the increase in the throughput at low values of *ω*. On the other hand, for *N*_*p*_ = 5, the impact of channel uncertainty is significant and large amount of extra power is needed during the re-optimization. Here, for small value of *ω* i.e., 0.001, the central node allocates less power, which may not suffice to provide successful decoding. Thus, the central node may perform re-optimizations multiple times, each re-optimization followed by retransmissions from the nodes. These retransmission cause the decrease in the throughput for *ω* = 0.001. This phenomena is further explained in Fig 8 for SNR $1/\sigma_{n}^{2}=-5$ dB. This figure plots the average throughput vs. *ω*. The difference between *N*_*p*_ = 200 and *N*_*p*_ = 5000 is negligible. It is clear that as the value of *ω* increases the throughput increases until a specific point. Beyond this point the throughput starts decreasing. These points are marked with black dots. This behaviour is due to the reason that initially for small values of *ω* in each re-optimization small power is allocated by the central node. Each time this power does not suffice to provide successful decoding at the destination. This requires multiple re-optimization and consequently multiple retransmissions, which causes low throughput. As *ω* increases more and more power is allocated which decreases the need of multiple retransmissions. However, beyond the optimal point so much high power is allocated that causes the activation of more relays. This activation of more relays drive again the throughput downwards.

It can also be observed that as *N*_*p*_ increases these points move to the left. This can be explained by the fact that as we increase the quality of the channel estimation less power is needed to result in successful decoding at the destination.

This figure also demonstrates that at these optimal points the throghputs for *N*_*p*_ = [5, 15, 45, 90, 200] are [37%, 21%, 9.37%, 6.25%, 3.125%] less than that of *N*_*p*_ = 5000.

**Fig 9:**
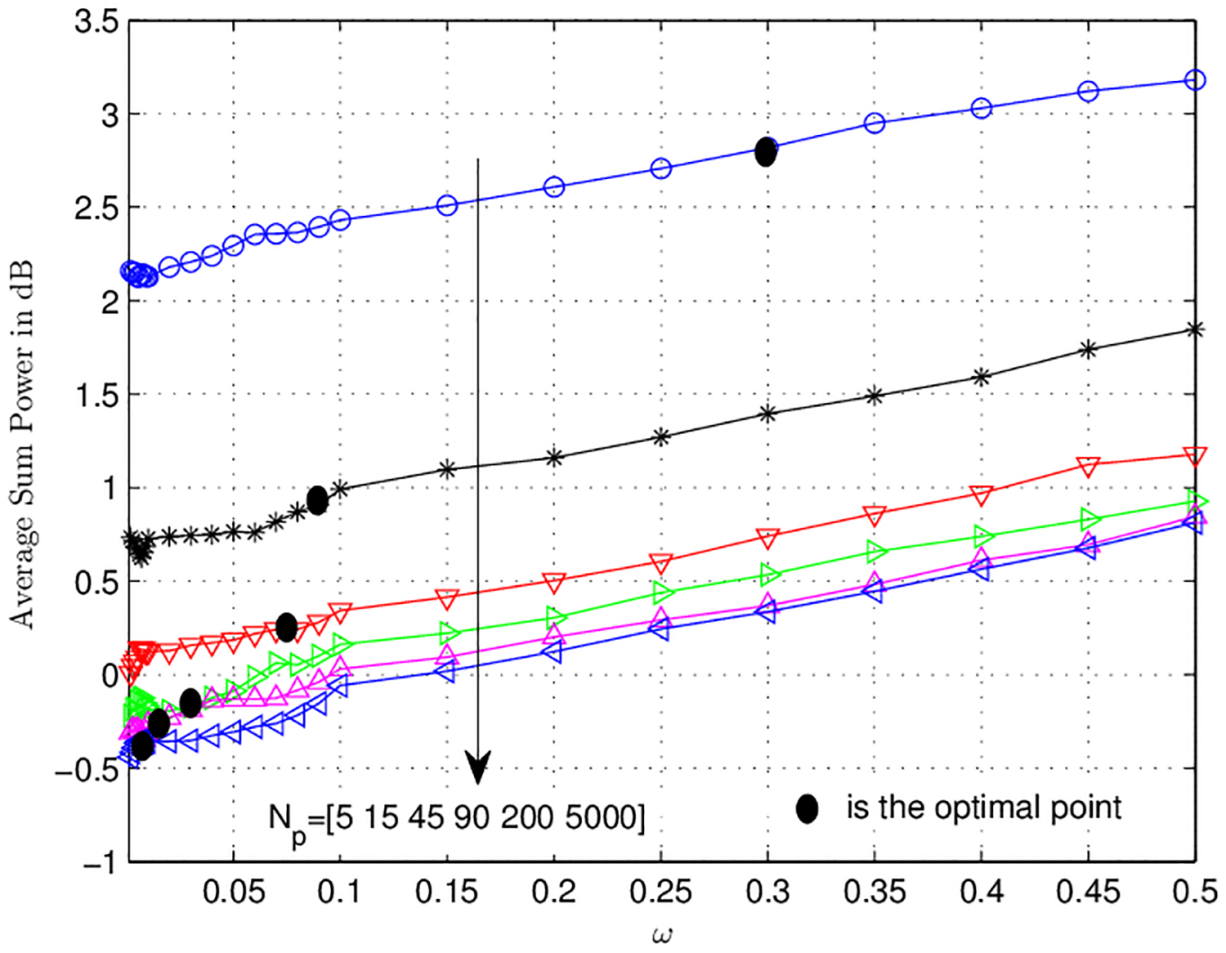
Average sum power consumed (in dB) curves vs. trade-off factor *ω* are depicted. The variable *N*_*p*_ denotes the number of pilot symbols used for estimation.

Fig 9 depicts the average sum power consumed over *ω* at SNR $1/\sigma_{n}^{2}=-5$ dB. The optimal points corresponding to Fig 8 are again plotted here. In cotrast to Fig 8, the power consumption increases as the value of *ω* is increased. This is intuitive, because, for high value of *ω*, the central node will allocate more extra power to the nodes in order to decode successfully at the destination.

At the optimul points the difference between the power consumed for *N*_*p*_ = 5000 and *N*_*p*_ = [5, 15, 45, 90, 200] is [3 dB, 1.5 dB, 0.75 dB, 0.25 dB, 0.1 dB].

## 9 Conclusion

Several power allocation algorithms with different objectives have been developed in the literature for relay network. These contributions consider perfect channel knowledge. Perfect channel knowledge is not viable in practice. This paper presents a framework to analyse the impact of imperfect channel knowledge on power allocation in conjunction with ARQ. The simulation results show that regarding sum power consumption, the power allocation algorithm under consideration with the devised strategy is robust to the channel uncertainties.

## Supporting information

S1 File Excel file containing values for average consumed power in dB, outage, and throughput with the respective SNR values from -8 dB to 2 dB with different number of pilot symbols. (XLSX)

S2 File Excel file containing values for average consumed power in dB and throughput with the respective Omega values from 0.001 to 0.5 with different number of pilot symbols and SNR -5 dB. (XLSX)
